# Dislocation-Based CPFEM and Phase-Field Study on the Stress Corrosion Cracking of Randomly Textured Magnesium Alloys

**DOI:** 10.3390/ma19102051

**Published:** 2026-05-14

**Authors:** Xu Zhai, Chao Xie, Xuedao Shu, Yupeng Liu

**Affiliations:** 1 The Faculty of Mechanical Engineering and Mechanics, Ningbo University, Ningbo 315211, China; nbuzhai_xu@163.com (X.Z.); shuxuedao@nbu.edu.cn (X.S.); 2 Meitaike Precision Manufacturing (Ningbo) Co., Ltd., Ningbo 315800, China; mtkyupeng_liu@163.com

**Keywords:** polycrystalline magnesium, stress corrosion cracking, dislocation density, crystal plasticity, phase-field model

## Abstract

Magnesium (Mg) alloys are promising for automotive lightweighting and the low-altitude economy, yet their reliability is challenged by stress corrosion cracking (SCC). To realize a quantitative and physics-based evaluation of SCC resistance, this study develops a mesoscale simulation framework coupling dislocation density-based crystal plasticity with an anodic dissolution phase-field model. A 2D representative volume element is constructed for randomly textured polycrystalline Mg to investigate the synergistic acceleration of corrosion by dislocation slip and hydrostatic stress. Results show that heterogeneous dislocation multiplication induced by pre-deformation is the decisive factor in corrosion path selection. In soft-oriented grains, high dislocation densities elevate the interface kinetic coefficient to levels substantially higher than those in hard-oriented regions. Notably, within such soft grains, the contribution of dislocation density to the interface kinetic coefficient can be up to 7.7 times that of hydrostatic stress, establishing dislocation-induced lattice disorder as the primary accelerator for transgranular corrosion. Hard-oriented grains effectively impede corrosion propagation due to restricted dislocation proliferation. This study elucidates how grain orientation-dependent dislocation evolution regulates corrosion morphology, revealing that the random texture delays overall structural failure based on a “weakest-link” mechanism.

## 1. Introduction

Magnesium (Mg) alloys exhibit significant potential for applications in automotive lightweighting and the emerging “low-altitude economy” due to their high specific strength and low density [1,2,3,4]. However, poor corrosion resistance remains a primary bottleneck restricting their large-scale industrial adoption [5]. Particularly under service loads, Mg alloys are highly susceptible to environmentally assisted degradation, specifically stress corrosion cracking (SCC). This phenomenon poses a severe threat to structural integrity and limits the broader implementation of Mg-based components [6,7].

The phase-field method (PFM) has emerged as a powerful continuum mechanics-based approach for simulating interface evolution. By introducing a continuous order parameter to describe material interfaces implicitly, PFM bypasses the complexities associated with explicit interface tracking required by traditional methods [8]. Sofiani et al. [9] utilized PFM to simulate corrosion pit evolution in low-carbon steel, demonstrating its efficacy in capturing complex morphological changes. Mai et al. [10] developed a phase-field model for pitting-induced SCC, successfully capturing the transition from pitting to crack propagation by correlating the interface kinetic coefficient with the crack-tip stress field. Compared to conventional techniques, PFM offers distinct advantages in multi-field coupled simulations due to its ability to seamlessly integrate mechanical stress fields and mass transport through differential equations [11].

In the field of mesoscale simulation, the Representative Volume Element (RVE) method is a well-established approach for establishing microstructure-property relationships in polycrystalline materials. The crystal plasticity finite element method (CPFEM) is a numerical technique that couples crystal plasticity theory with the finite element method to predict the mechanical behavior of crystalline materials across scales from grains to macroscopic structures. Zhu et al. [12] utilized a multiscale framework integrating CPFEM with a periodic homogenization strategy to systematically examine the influence of RVE size and microstructural features on the prediction of ductility limits in polycrystalline aggregates. Their work validated the efficacy of the RVE approach in analyzing the representativeness of mesomechanical responses. Jalili et al. [13] utilized a 3D full-field crystal plasticity computational homogenization method to construct realistic RVE models of the AZ31 Mg alloy consisting of 34 to 60 grains, revealing how the spatial distribution of basal and non-basal grain orientations regulates the mechanisms underlying strain localization. Extending the RVE method to the domain of corrosion simulation allows for the microscale capture of how mesoscale microstructural features, such as grain orientation and dislocation multiplication, regulate localized corrosion activity and the subsequent damage evolution pathways.

When Mg alloys are subjected to mechanical loading, stress significantly accelerates the corrosion process. To quantify this mechano-chemical coupling, various mechanical models have been integrated into PFM frameworks. Mathew et al. [14] proposed a coupled crystal plasticity phase-field model that links anisotropic mechanical response with electrochemical dissolution to simulate SCC, revealing the impact of grain orientation and pit morphology on crack propagation. Kandekar et al. [15] introduced a bi-directionally coupled phase-field framework based on the preCICE open-source library, considering the acceleration of the corrosion front due to plasticity. However, existing research faces limitations: the model by Mathew et al. [14] relies on a power-law hardening rule without incorporating dislocation density as a state variable, while the study by Kandekar et al. [15] employs an anisotropic elastic constitutive law, which fails to accurately account for the contributions of dislocation multiplication.

Extensive research indicates that dislocation plays a critical role in SCC. The classic Gutman model [16] posits that dislocation multiplication enhances local distortion energy and electrochemical potential, thereby accelerating anodic dissolution. Liu et al. [17] demonstrated that high-density dislocations introduced by pre-deformation act as preferential corrosion channels, significantly accelerating localized corrosion. Furthermore, Zhao et al. [18] found that increased stress leads to higher dislocation densities, where slip-band-induced micro-channels serve as rapid pathways for corrosive media to penetrate the matrix. Additionally, high local hydrostatic stress within the crystal expands lattice interstices, facilitating the ingress of corrosive species and further accelerating degradation [19]. Consequently, incorporating physics-based dislocation density evolution equations into the simulation framework, as opposed to relying on simplified power-law hardening criteria, is a necessary prerequisite for accurately characterizing the stress corrosion behavior of Mg alloys.

In response to the SCC behavior of randomly textured polycrystalline Mg alloys, this study develops a simulation framework coupling dislocation-based crystal plasticity with anodic dissolution phase-field modeling. By incorporating dislocation density evolution equations to describe the accumulation of crystal defects induced by plastic deformation, and coupling hydrostatic stress effects into the interface kinetic coefficient, this framework achieves a quantitative characterization of the synergistic acceleration mechanism between dislocation slip and hydrostatic stress. Through numerical simulation and quantitative analysis, this study elucidates the mechanisms by which hydrostatic stress and dislocation multiplication co-regulate corrosion path evolution and damage rates.

## 2. Methodology

### 2.1. Crystal Plasticity Model at Finite Strains

#### 2.1.1. Kinematics and Constitutive Relationship

The total deformation gradient ***F*** is multiplicatively decomposed into an elastic part ***F***_e_ and a plastic part ***F***_p_:

(1) $$F=F_{e}\cdot F_{p},$$

where ***F***_p_ represents the deformation gradient due to plastic shear, and ***F***_e_ corresponds to the elastic stretching and rigid rotation of the lattice.

In the intermediate configuration, the elastic Green-Lagrange strain ***E***_e_ is defined as:

(2) $$E_{e}=\frac{1}{2}\left(F_{e}^{T}\cdot F_{e}-I\right),$$

where ***F***_e_ is obtained from Formula (1), and ***I*** is the second-order identity tensor.

The Mandel stress ***S*** is determined by:

(3) $$S=D(\varphi )C:E_{e},$$

where ***C*** is the elastic stiffness tensor of the lattice in the global coordinate system, and *D*(*φ*) = *φ*^2^ is the stiffness degradation function dependent on the Phase-field order parameter *φ*.

#### 2.1.2. Dislocation Density-Based Model

The plastic part of the velocity gradient ***L***_P_ is related to the rate of the plastic deformation gradient ***F***_p_ and the shear rate of each slip system as follows:

(4) $$L_{P}={\dot{F}}_{P}\cdot F_{P}^{-1}=\overset{N_{s}}{\underset{\alpha =1}{\sum }}{\dot{\gamma }}^{\left(\alpha \right)}m^{\left(\alpha \right)}⊗n^{\left(\alpha \right)},$$

where ***m***^(*α*)^ and ***n***^(*α*)^ represent the unit vectors for the slip direction and the slip plane normal of the *α*-th slip system, respectively. *N*_s_ denotes the total number of active slip systems (*N*_s_ = 12 in this study), comprising three basal, three prismatic, and six pyramidal <c + a> slip systems.

The shear rate on each slip system is given by the Orowan equation [20]:

(5) $$\dot{\gamma }=\dot{\gamma_{0}}exp\left[-\frac{Q_{a}}{k_{b}T}\left\{1-{\left(\frac{\left|\tau_{eff}\right|}{\tau_{p}}\right)}^{p}\right\}\right]sign(\tau ),$$

where $\dot{\gamma_{0}}$ is the reference shear strain rate, *Q*_a_ is the activation energy to overcome obstacles, *k*_b_ is the Boltzmann constant, *T* is the absolute temperature, *τ*_eff_ is the effective resolved shear stress, *τ*_p_ is the Peierls stress, and *p* is a fitting parameter associated with the glide resistance profile.

The effective shear stress *τ*_eff_ is obtained by subtracting the passing stress *τ*_pass_, which represents the obstacle resistance to dislocation motion, from the resolved shear stress *τ*, i.e.,:

(6) $$\tau_{eff}=\left\{\begin{array}{c} |\tau |-\tau_{pass}\;\;\;\;for\;\;\;\;|\tau |>\tau_{pass}\\ 0\;\;\;\;\;\;\;\;\;for\;\;\;\;|\tau |\leq \tau_{pass} \end{array}\right.,$$

where *τ* is the resolved shear stress on the slip system.

The passing stress τpass on the α-th slip system is calculated as:

(7) $$\tau_{pass}^{\alpha }=D(\varphi )\left\{\underset{Forest\;dislocation\;effect}{\underbrace{Gb{\left(\underset{\alpha^{\prime }=1}{\overset{N_{s}}{\sum }}\xi_{\alpha \alpha^{\prime }}\left(\rho_{m}^{\alpha^{\prime }}+\rho_{d}^{\alpha^{\prime }}\right)\right)}^{\frac{1}{2}}}}+\underset{Hall-Petch\;effect}{\underbrace{\frac{k_{y}}{\sqrt{d}}}}\right\},$$

where *G* is the shear modulus, *b* is the length of the Burgers vector, *ξ_αα_*_′_ denotes the interaction matrix between the slip system *α* and *α*′, *k*_y_ is the Hall-Petch coefficient, and *d* is the equivalent grain diameter. *ρ*_d_ is the dislocation dipole density, *ρ*_m_ is the mobile dislocation density. The evolution equations for dislocation density are adopted from [20].

### 2.2. Anodic Dissolution Phase-Field

The theoretical foundation of the phase-field method originates from irreversible thermodynamics [21,22]. For the corrosion process, the Allen-Cahn equation is employed to describe the evolution of the order parameter:

(8) $$(\nabla \cdot \zeta -\omega )-\frac{1}{L}\frac{\partial \varphi }{\partial t}=0,$$

where *L* is the interface kinetic coefficient. The negative damage driving force *ω* is defined as the variational derivative of the system free energy with respect to the phase-field order parameter *φ*. It quantifies the thermodynamic driving force that governs the evolution of the solid/liquid interface during corrosion. The designation “negative” reflects the fact that the direction of this driving force aligns with the direction of decreasing system free energy, i.e., *ω* = −δ*Ψ*/δ*φ*, where *Ψ* is the free energy density. The negative damage driving force *ω* and the negative damage flux ***ζ*** are conjugate to the phase-field *φ* and the phase-field gradient **∇***φ*, respectively.

The expression for the negative damage driving force *ω* is expressed as follows:

(9) $$\;\omega =-2A[c-h_{1}(\varphi )(c_{se}-c_{Le})-c_{Le}](c_{se}-c_{Le})h_{1}^{\prime }(\varphi )+wg^{\prime }(\varphi ),$$

where *w* is the height of the double-well potential function g(*φ*) = *φ*^2^(1 − *φ*)^2^. The double-well potential function satisfies g(*φ* = 0) = 0 and g(*φ* = 1) = 0. To meet the electrochemical requirements, ℎ_1_(*φ*) = −2*φ*^3^ + 3*φ*^2^ is used in the model, and a similar free energy density curvature *A* is defined for both the solid and liquid phases. *c*_se_ and *c*_Le_ can be derived from the equilibrium concentration *c*_solid_ in the metal and the saturation concentration *c*_sat_ in the liquid phase, where *c*_se_ = *c*_solid_/*c*_solid_ = 1 is the normalized equilibrium concentration of the solid, and *c*_Le_ = *c*_sat_/*c*_solid_ is the normalized equilibrium concentration of the liquid.

The negative damage flux ***ζ*** is expressed as follows:

(10) $$\zeta =G_{c}l_{c}\nabla \varphi ,$$

where *G*_c_ is the surface energy density, and *l*_c_ is the interfacial characteristic length.

The interface kinetic coefficient *L* is expressed as follows:

(11) $$\;L=L_{0}exp\left(\frac{\sigma_{m}V_{m}}{RT}\right)\left(\frac{\rho^{total}}{\rho_{0}^{total}}\right),$$

where *L*_0_ is the reference interfacial kinetic coefficient, *σ*_m_ is the hydrostatic stress, *V*_m_ is the molar volume of Mg, *R* is the gas constant, and *T* is the absolute temperature. *ρ*^total^/*ρ*_0_^total^ reflects the acceleration effect of lattice distortion on anodic dissolution.

### 2.3. Mass Transfer Field

The expression for the mass transfer field in the model is [23]:

(12) $$\frac{\partial c}{\partial t}-\nabla \cdot M(\nabla [c-h_{1}(\varphi )(c_{se}-c_{Le})-c_{Le}])=0$$

where *c* is the dimensionless concentration of Mg ions, *M* is the diffusion coefficient. This expression links the concentration gradient-driven diffusion to the variation in chemical potential at the phase interface, thereby guaranteeing the thermodynamic consistency of mass transfer with the evolving interface.

### 2.4. RVE Configuration and Boundary Conditions

**Figure 1:**
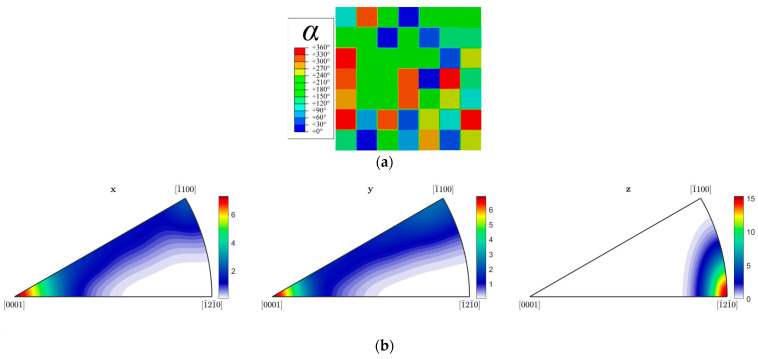
(**a**) Geometric model of the 2D regular grain RVE (7 × 7 grains, 49 μm × 49 μm); (**b**) IPF of the RVE model.

All finite element simulations are carried out using Abaqus (version 6.14, Dassault Systèmes, Providence, RI, USA). To investigate the stress-corrosion coupling behavior of randomly textured polycrystalline Mg that is extensively utilized as integrated components in automobile and low-altitude economy industry based on die-casting technologies, a 2D RVE model, as shown in Figure 1a, is constructed. This 2D simplification significantly enhances computational efficiency while facilitating the intuitive tracking of the dynamic evolution of corrosion pathways. The model comprises a 7 × 7 array of square grains, with each individual grain measuring 7 μm × 7 μm, resulting in a total domain size of 49 μm × 49 μm. A planar random texture is assigned to the model, with Euler angles defined as (α, 90°, 0°), where *α* is uniformly distributed within the range of [0°, 360°]. Figure 1b presents the inverse pole figure (IPF) of the crystal orientations for this regular grain model. In the leftmost Z-axis IPF, the pole density is sharply concentrated at the [−12–10] direction, indicating that all grains are oriented with their [−12–10] direction parallel to the Z-axis. Because [−12–10] is crystallographically perpendicular to the c-axis [0001], this implies that the c-axes of all grains lie in the X–Y plane. Furthermore, the distribution patterns in the X and Y IPFs are nearly identical, and the intensities associated with the c-axis [0001] are comparable. Consequently, the c-axes exhibit no preferred orientation within the X–Y plane, confirming a planar random texture.

It should be noted that deformation twinning, a well-recognized deformation mechanism in Mg alloys, is not explicitly incorporated in the present crystal plasticity formulation. The rationale is twofold. The Mg alloy components under investigation are typically manufactured by integrated die-casting, which yields a fine and uniform grain structure. The mean grain size in the model is approximately 7 μm, falling within the fine-grained regime where twinning activity is significantly suppressed, as grain boundaries effectively hinder twin nucleation and propagation, rendering dislocation slip the dominant deformation mode. Should the framework be extended to coarse-grained microstructures or larger pre-strain levels, the role of twin boundaries as obstacles to dislocation motion and as potential preferential corrosion channels would need to be explicitly accounted for.

**Figure 2:**
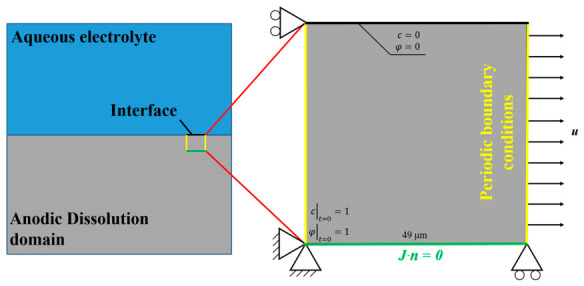
Boundary conditions of stress corrosion RVE model.

The boundary conditions for the stress-corrosion coupled RVE model are depicted in Figure 2. At the initial state, the phase-field order parameter *φ* and the solute concentration *c* are set to unity throughout the domain. Regarding mechanical loading, a uniaxial tensile displacement load is applied along the x-direction of the RVE at a constant strain rate of 10^−3^ s^−1^. For the environmental simulation, considering that the corrosive medium infiltrates only from the upper surface, Dirichlet boundary conditions (represented by the black line) are applied exclusively to the top boundary of the RVE. This setup simulates the actual service conditions of a specimen exposed to a 3.5 wt.% NaCl solution. To prevent the artificial introduction of non-physical “top-to-bottom” penetration paths, periodic boundary conditions are not employed in the y-direction (vertical). Instead, periodic boundary conditions for both the phase-field and mass transfer fields are prescribed on the left and right boundaries (yellow lines), while the bottom boundary (green line) is assigned a zero-flux condition defined by ***J***·***n*** = **0**, where ***J*** is the diffusion flux of the corrosive species (Mg^2+^ ions) and ***n*** is the outward unit normal vector. This condition ensures that no mass transfer occurs across the bottom boundary, so that the corrosive medium enters exclusively from the top free surface, consistent with the actual service condition in which only the upper surface of the specimen is exposed to the corrosive solution.

## 3. Results

### 3.1. Micromechanical State After Pre-Straining

Prior to the corrosion evolution simulation, a uniaxial tensile pre-strain of 0.7% is applied to the RVE model, followed by a 10 s stress relaxation period. This procedure establishes the initial micromechanical response and dislocation multiplication state. This specific strain level is chosen to reflect the typical service conditions of die-cast integrated Mg alloy components. Such components are generally characterized by fine grains and a near-random texture, and they experience limited deformation as non-primary load-bearing structural elements. Consequently, the 0.7% pre-strain effectively induces dislocation multiplication representative of practical scenarios without triggering excessive plastic flow that could distort the geometric configuration.

**Figure 3:**
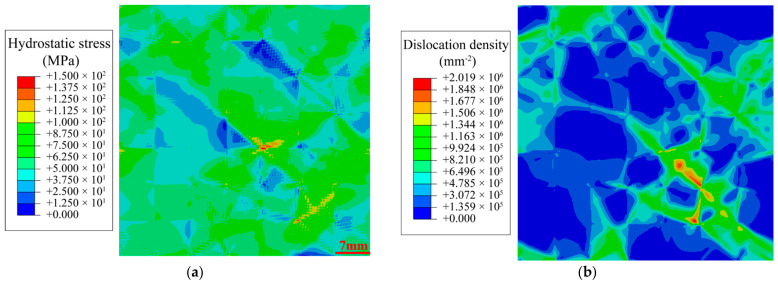
Micromechanical state of the RVE after 0.7% pre-strain and 10 s relaxation: (**a**) Hydrostatic stress distribution; (**b**) Dislocation density distribution.

As shown in Figure 3, the pre-straining dislocation density exhibits a pronounced dependence on grain orientation. In soft-oriented grains, dislocations proliferate extensively, forming localized high-density regions. Conversely, in hard-oriented grains, the dislocation density remains low due to restricted deformation compatibility. In contrast to the grain-interior dislocation distribution, the hydrostatic stress primarily concentrates at the triple junctions and grain boundaries.

### 3.2. Dislocation Heterogeneity and Transgranular Corrosion Kinetics

**Figure 4:**
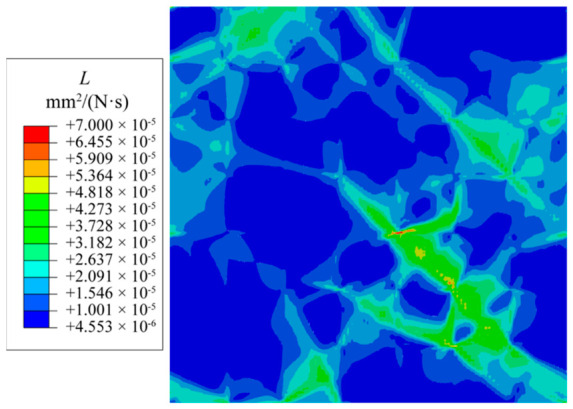
Distribution of the interface kinetic coefficient *L*.

Figure 4 illustrates the distribution of the interface kinetic coefficient, *L*, within the pre-strained RVE. In soft-oriented grains with significant dislocation proliferation, *L* reaches approximately 1 × 10^−4^ mm^2^/(N·s). In contrast, in hard-oriented grains where deformation is restricted, the value drops below 1.5 × 10^−5^ mm^2^/(N·s).

**Figure 5:**
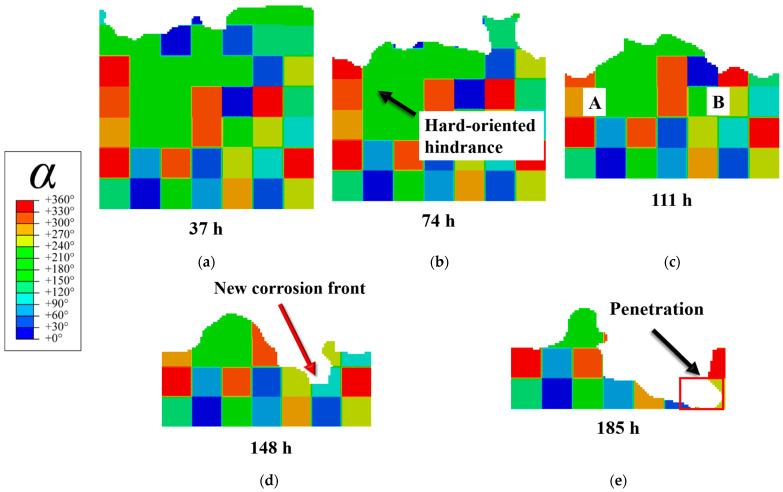
Temporal evolution of corrosion morphology: (**a**) 37 h; (**b**) 74 h; (**c**) 111 h, A and B indicate grains at the left and right corrosion fronts; (**d**) 148 h; (**e**) 185 h.

The corrosion morphology evolution shown in Figure 5 further substantiates the kinetic discrepancies among grains of different orientations. In Figure 5a, corrosion propagates preferentially within the soft-oriented grains (the left side near the upper interface), exhibiting a characteristic transgranular corrosion mode. By the stage shown in Figure 5b, the soft-oriented grains neighboring the hard-oriented region on the left are largely dissolved, and further propagation of corrosion into the hard-oriented region becomes impeded (black arrows).

Subsequently, at the stage captured in Figure 5c, the left and right corrosion paths reach comparable depths. Thereafter, the left path ceases to propagate significantly, whereas the right path continues to advance and eventually exceeds the left side in Figure 5d. Finally, at 185 h (Figure 5e), complete corrosion penetration occurs on the right side of the model.

### 3.3. Corrosion Regulation via Dislocation Evolution

**Figure 6:**
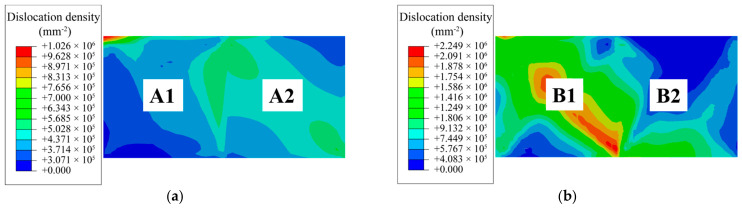

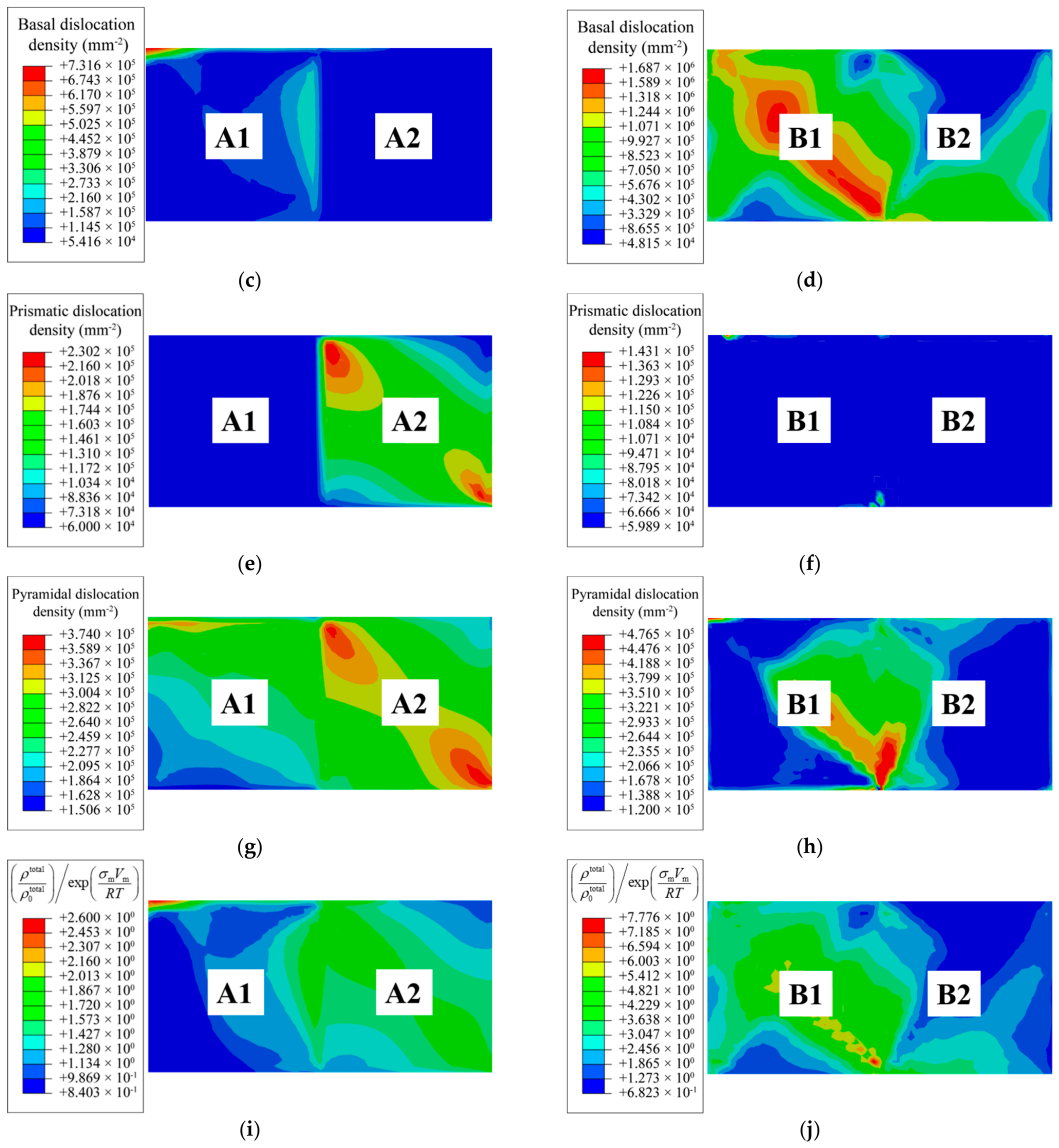
Quantitative comparison of hard-oriented (A1, A2) and soft-oriented (B1, B2) grains: (**a**,**b**) Total dislocation density; (**c**,**d**) Basal dislocation density; (**e**,**f**) Prismatic dislocation density; (**g**,**h**) Pyramidal dislocation density; (**i**,**j**) Ratio of the contribution of dislocation density to hydrostatic stress in the interface kinetic coefficient *L*.

To further validate how grain orientation regulates corrosion kinetics through dislocation evolution, a quantitative comparative analysis is performed on the left and right corrosion fronts at the identical depth shown in Figure 5c. The left hard-oriented region includes grains A1 and A2, while the right soft-oriented region includes grains B1 and B2. The total dislocation density, activation of slip systems, and the relative contributions to the interface kinetic coefficient for these four grains are presented in Figure 6.

Figure 6a,b shows the total dislocation density. Compared with the hard-oriented grains on the left side (A1 and A2), the soft-oriented grains on the right side (B1 and B2) exhibit substantially higher maximum dislocation densities: the B1/A1 ratio is approximately 2.5, and the B2/A2 ratio is approximately 4.8.

Figure 6c–h presents comparisons of the basal, prismatic, and pyramidal dislocation densities for grains on the two sides, respectively. In grains A1 and A2, non-basal dislocations are predominant, accounting for approximately 72% and 75% of the total dislocation density, respectively. This indicates that non-basal slip systems dominate the deformation in these grains. In contrast, grains B1 and B2 exhibit a clear predominance of basal dislocations, which constitute approximately 70% and 63% of the total density, respectively.

Figure 6i,j shows the contribution ratio of the interfacial kinetic coefficient. By decoupling the dislocation density and hydrostatic stress terms within the interfacial kinetic coefficient *L*, their relative contributions are quantified. In the hard-oriented region (Figure 6i), the contribution ratio (dislocation/hydrostatic) is approximately 2, indicating that both mechanisms competitively influence the corrosion acceleration process. Hydrostatic stress tends to trigger brittle cracking by promoting the nucleation and propagation of micro-voids or cleavage cracks; conversely, dislocation multiplication accommodates plastic deformation through sustained slip activity. In the soft-oriented region (Figure 6j), the maximum ratio reaches 7.7 for grain B1 and approximately 3.6 for grain B2. Within these grains, the contribution of the dislocation density field significantly outweighs that of the hydrostatic stress field, establishing dislocation-induced lattice disorder as the primary driver for accelerated corrosion.

The activation of basal slip in soft-oriented grains markedly elevates the overall dislocation density, thereby amplifying crystalline disorder and ultimately accelerating corrosion propagation. The corrosion morphologies and dislocation density distributions presented in Figure 5 and Figure 6 provide evidence for this mechanism: grains A1 and A2, which are dominated by non-basal slip, exhibit significantly lower dislocation densities and a correspondingly delayed corrosion progression. Conversely, grains B1 and B2, primarily characterized by basal slip, accumulate higher dislocation densities and undergo more rapid dissolution. This direct correlation demonstrates that lattice defect disparities, dictated by crystal orientation, constitute the primary factor driving the differentiation of corrosion activity among grains.

### 3.4. Mass Loss and Corrosion Depth Evolution

**Figure 7:**
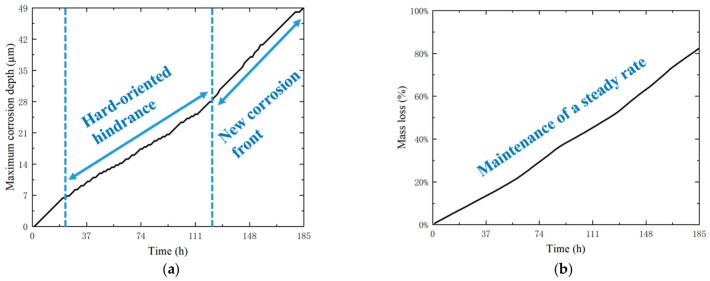
(**a**) Maximum corrosion depth vs. time; (**b**) Cumulative mass loss rate vs. time.

This section provides an evaluation of the driving role of heterogeneous dislocation distribution in stress corrosion damage, focusing on two metrics: maximum corrosion depth and cumulative mass loss rate. The results are summarized in Figure 7.

Figure 7a illustrates the evolution of the maximum corrosion depth over time. During the initial stage (0~20 h), the corrosion depth increases rapidly within the soft-oriented grains. Subsequently (20~120 h), the expansion rate slows down significantly as the corrosion front encounters hard-oriented grains characterized by low dislocation density. This plateau corresponds to the stage shown in Figure 5b, where the corrosion path on the left side is obstructed. From 120 h onward, the corrosion initiates a new propagation path through a soft-oriented region on the opposite side of the model. Consequently, the maximum corrosion depth becomes dominated by the right side (Figure 5d), leading to a renewed acceleration in the expansion rate until complete penetration occurs at 185 h. Figure 7b shows the cumulative mass loss rate as a function of time. In contrast to the fluctuating depth expansion rate, the mass loss rate remains remarkably stable throughout the simulation. Hard-oriented grains arrest depth propagation locally, yet lateral pit growth in adjacent soft grains sustains a steady active interface length, resulting in a near-linear mass loss rate.

In conclusion, in a random texture, hard-oriented grains, in which dislocation multiplication is unfavorable, act as effective barriers that impede the inward propagation of corrosion paths. In accordance with a “weakest-link” mechanism, this barrier effect delays catastrophic structural failure.

### 3.5. Comparison with Existing Models and Implications

To clarify the advancement of the present framework, it is instructive to compare it with recently developed coupled crystal plasticity–phase-field models for SCC. Mathew et al. [14] proposed a model linking anisotropic mechanical response to electrochemical dissolution; however, the constitutive description relied on a power-law hardening rule without introducing dislocation density as an internal state variable. Consequently, the evolution of lattice defects and their contribution to corrosion kinetics could not be explicitly captured. Kandekar et al. [15] established a bi-directionally coupled phase-field framework based on the preCICE open-source library, yet the mechanical model was restricted to an anisotropic elastic constitutive law, neglecting the effects of dislocation multiplication. This represents a notable simplification, given that extensive research has demonstrated dislocation multiplication to be the dominant factor accelerating SCC. The present model incorporates physically based dislocation density evolution equations within the crystal plasticity formulation to explicitly describe dislocation multiplication. Both the resulting dislocation density field and the hydrostatic stress field are directly coupled into the interface kinetic coefficient, thereby enabling a quantitative decoupling of the individual contributions from dislocation-induced lattice disorder and stress-driven mechanical effects. Building upon this physics-based description, the model further realizes numerical simulation of SCC in polycrystalline systems at the grain scale by integrating CPFEM with the anodic dissolution phase-field model. This provides a mesoscale analysis tool with more clearly resolved physical mechanisms for evaluating SCC resistance.

## 4. Conclusions

In this study, a mesoscale simulation framework for SCC governed by crystal defects is developed for randomly textured fine-grained Mg alloys. The framework is based on a coupled dislocation-based crystal plasticity and anodic dissolution phase-field algorithm. By accounting for the amplification effect of dislocation multiplication and hydrostatic stress on corrosion current density following pre-straining, the SCC behavior of Mg alloys is successfully captured. The primary conclusions are as follows:

- Heterogeneous dislocation multiplication induced by pre-deformation dictates the selection of transgranular corrosion paths. High-density dislocation zones within soft-oriented grains act as preferential dissolution sites, forming dominant transgranular corrosion paths. When dominant corrosion paths encounter hard-oriented grains, where dislocation is restricted, propagation is locally retarded. Although corrosion eventually circumvents these obstacles by initiating new channels in adjacent soft-oriented regions, the overall structural failure is delayed, reflecting a “weakest-link” characteristic.
- Dislocation density constitutes the dominant contributor to accelerated corrosion kinetics under the conditions examined. Analysis of the contribution ratio of the interfacial kinetic coefficient reveals that, within the soft-oriented grains analyzed (B1 and B2), the contribution of the dislocation density field to the local corrosion driving force is up to approximately 7.7 times that of the hydrostatic stress field. In the hard-oriented region (A1 and A2), the two contributions are of comparable magnitude, with a ratio of approximately 2.
- Soft-oriented grains exhibit a predominance of basal dislocations, which constitute approximately 70% and 63% of the total density in grains B1 and B2, respectively, and are associated with elevated overall dislocation densities and accelerated corrosion propagation. Conversely, grains dominated by non-basal dislocations display markedly lower total dislocation densities and correspondingly delayed corrosion. This correlation demonstrates that the differentiation of corrosion activity among grains is fundamentally governed by crystal orientation through its influence on the operative slip systems and the resulting disparities in accumulated lattice defects.

These findings establish a quantitative, micro-mechanism-informed link between dislocation-based plasticity and macroscopic SCC susceptibility, offering a mesoscale pathway for the texture-informed regulation of SCC in Mg alloys.

## Data Availability

The original contributions presented in this study are included in the article. Further inquiries can be directed to the corresponding author.
